# Superior Mesenteric Artery Syndrome in a Low-Resource Setting: Case Series Highlighting a Rare Etiology of Intestinal Obstruction

**DOI:** 10.1155/cris/7930036

**Published:** 2025-10-29

**Authors:** Abate Bane Shewaye, Zebeaman Tibebu Gorfu, Kaleb Assefa Berhane, Megersa Regassa, Amsalework Daniel Fanta, Fekadu Ayalew

**Affiliations:** ^1^Department of Internal Medicine, College of Health Sciences, Addis Ababa University, Addis Ababa, Ethiopia; ^2^Department of Internal Medicine, Adera Medical and Surgical Center, Addis Ababa, Ethiopia; ^3^Department of Surgery, Adera Medical and Surgical Center, Addis Ababa, Ethiopia; ^4^Department of Radiology, Adera Medical and Surgical Center, Addis Ababa, Ethiopia

**Keywords:** case series, Ethiopia, small bowel obstruction, superior mesenteric artery (SMA) syndrome

## Abstract

Superior mesenteric artery (SMA) syndrome is an extremely rare condition that can result from a multitude of causes that devoid the mesenteric fat pad or structurally narrow the space resulting in duodenal obstruction. It is predominantly seen in females. If not treated, it can result in life-threatening complications. Herein, we present four patients with SMA syndrome who presented to our outpatient department at Adera Medical and Surgical Center, Addis Ababa, Ethiopia.

## 1. Introduction

Superior mesenteric artery (SMA) syndrome is a rare disease that can go unrecognized and undiagnosed. It was first described 160 years ago by Rokitansky C in anatomy text under the name “internal hernias” that led to incarceration [1]. However, the first comprehensive description was given by Wilkie on a case series of 75 patients in 1927 [2]. Over the years, several cases of SMA syndrome have been reported. However, its exact incidence and prevalence remain unknown. It is prevalent in 0.3% of patients with abdominal pain and 0.013 % of consecutive upper gastrointestinal (GI) series [3, 4]. It can affect all age groups, most commonly affecting those between 10 and 39 years of age, with female predominance. No racial differences have been identified so far [5].

SMA syndrome is characterized by a decrease in the aortomesenteric angle (AMA). It has diverse causes. Its association with weight loss has been established in cases of hypermetabolism (trauma, hyperthyroidism, and burns) [6], dietary conditions (anorexia nervosa and malabsorptive diseases) [7], and cachexia-causing conditions (acquired immune deficiency syndrome, cancer, and paraplegia) [8]. Congenitally short or hypertrophic ligament of Treitz, peritoneal adhesions, duodenal malrotation, Ladd's bands, abdominal aortic aneurysm, and mesenteric root neoplasm have also been identified in some cases [5]. It can be seen among patients with vertebral problems (lumbar hyper lordosis and surgical correction of scoliosis) [9]. Diagnosis is based on clinical symptomatology that is substantiated with radiographic evaluation. Its management is primarily conservative [8]. Surgical management is indicated only upon failure of medical intervention [10].

Reports of SMA syndrome are extremely scarce in Africa. In Ethiopia, there is only one report of two cases of SMA in two young Ethiopian female patients who showed symptomatic improvement upon surgical management (duodenojejunostomy) [11]. Here, we present a series of patients with SMA who were treated both medically and surgically (Table 1).

### 1.1. Case 1

**Figure 1:**
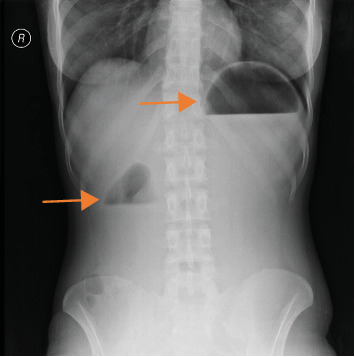
Plain abdominal X-ray showing dilated stomach and duodenal bulb with air–fluid level and paucity of gas in the remaining distal intestine.

A 23-year-old female patient from Addis Ababa presented to our outpatient department (OPD) with longstanding history of epigastric crampy pain, frequent bilious vomiting, and early satiety. She has lost 15 kgs over the past 3 years. The abdominal pain and vomiting improved with lying on left lateral position and taking fluid diet. Her symptoms were not improving despite repeated treatment for dyspepsia. Upon arrival, the patient was noted to be acutely sick looking, fatigued, and had frequent vomiting. Her vital signs were within the normal range, but her body mass index (BMI) was 16.4 kg/m^2^. Her buccal mucosa was dry, and she was emaciated. Her blood tests showed leukocytosis of 16,200/uL, hemoglobin (Hgb) 14 g/dL, and platelet count 363,000/uL with erythrocyte sedimentation rate (ESR) of 15 mm/h. She was hypokalemic with serum potassium level 3.0, and serum lipase was elevated to 1777 u/L (units per liter). However, renal function test (RFT) and liver function test (LFT) were normal. Her plain abdominal X-ray showed dilated stomach and duodenal bulb with air–fluid level and paucity of gas in the remaining distal intestine (Figure 1). Abdominal computed tomography (CT) scan showed-short aortomesenteric distance (AMD) (8 mm) and narrowed AMA (18°) with dilated stomach and proximal duodenum (1^st^, 2^nd^, and upper 3^rd^ portion). With the diagnosis of SMA syndrome and pancreatitis, the patient was managed with intravenous fluid, proton pump inhibitors, antiemetics, and potassium replacement. Three liters of bilious fluid was drained via nasogastric tube, and the patient got symptomatic relief. On 4^th^ day of admission, serum lipase level dropped to 440 u/L, she tolerated semisolid foods and was discharged improved. During follow-up visit after a week, she gained 3 kgs, had no vomiting or abdominal pain, and felt comfortable while lying on left lateral position. All the abnormal laboratory tests normalized as well.

### 1.2. Case 2

**Figure 2:**
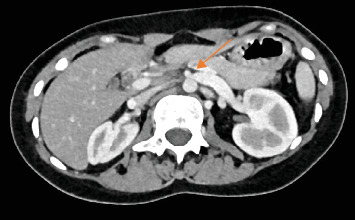
Dilated left renal vein with significant narrowing as it passes through aortomesenteric junction (Nut-cracker syndrome).

A 14-year-old female patient presented with worsening of postprandial projectile bilious vomiting with associated epigastric crampy abdominal pain of 1-month duration. She had a history of decreased appetite and lost 6 kg over the past 6 months. The symptoms have been present since childhood (past 8 years). She had been treated repeatedly for dyspepsia and gastroenteritis; however, the symptoms did not resolve. She did not have any stressor. At presentation her vitals were stable. Her BMI was 14.1 kg/m^2^. Scaphoid abdomen with mild epigastric tenderness was appreciated on physical examination. Upon investigation, leukocytosis with left shift was seen (white blood cell count [WBC] of 20,000 with 85.8% neutrophil). Serum lipase and amylase were both raised (serum lipase- 700, serum amylase- 199). ESR was also raised (45 mm/h.). RFT, LFT, and serum electrolyte were in the normal range. A short AMD (6 mm) and narrowed AMA (13°) with excessively dilated stomach and proximal duodenum (1^st^, 2^nd^, and upper 3^rd^ portion) were noted on abdominal CT scan. It further identified dilated left renal vein with significant narrowing as it passes through aortomesenteric junction (Nut-cracker syndrome) (Figure 2). Endoscopic evaluation revealed a distended stomach and duodenum with air insufflation. With the diagnosis of gastric outlet obstruction (GOO) secondary to distal duodenal obstruction secondary to SMA syndrome, the patient was admitted and prepared for surgery. Nasogastric tube decompression was done due to persistent vomiting. Intraoperatively, short SMA with acute angle significantly obstructing fourth part of duodenum was seen. An adhesive band surrounding the ligament of tretz was also identified. Stomach, first and second part of duodenum, was hugely dilated. Adhesion band release and duodenojejunal anastomosis were done. Postoperatively, the vomiting subsided. She was discharged after she tolerated oral feeding. Upon subsequent follow-up, the patient's symptoms had subsided, and her laboratory parameters had normalized.

### 1.3. Case 3

**Figure 3:**
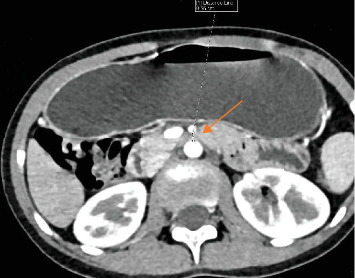
Abdominal CT scan showing a short AMD (5.6 mm).

A 29-year-old female patient presented to our OPD with recurrent history of postprandial vomiting of ingested matter and epigastric pain, with associated bloating and fatigue. She has a history of decreased appetite and weight loss of 10 kg over the last 6 months. Despite treatment for dyspepsia, her symptoms did not resolve. Upon evaluation, her vitals were in the normal range. Her BMI was 13.9 kg/m^2^. Mild epigastric tenderness was appreciated during abdominal examination. Complete blood count (CBC), serum electrolyte, LFT, RFT, serum amylase, serum lipase, and stool examination were all unremarkable. Urine human chorionic gonadotropin (HCG) test was negative. Abdominal CT scan revealed a short AMD (5.6 mm) (Figure 3) and narrowed AMA (15°) with dilated stomach and proximal duodenum (1^st^, 2^nd^, and upper 3^rd^ portion). With the diagnosis of SMA syndrome and functional dyspepsia, she was put on pantoprazole 40 mg per oral (PO) daily for 28 days, metoclopramide 10 mg PO nocturnal for 10 days and chlordiazepoxide and clidinium bromide 7.5 mg PO nocturnal for 1 month. She was advised on frequent feeds and sent home. On next follow-up, she gained 7 kg, and the vomiting had completely subsided.

### 1.4. Case 4

**Figure 4:**
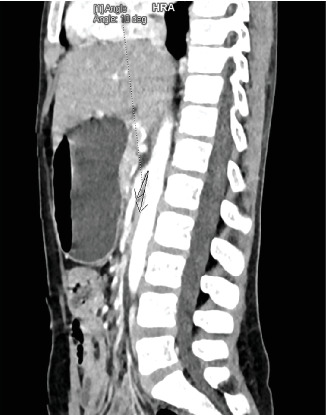
Abdominal CT scan showing narrow AMA (10°).

An 11-year-old female patient presented to our OPD with a 20-day history of periumbilical pain and postprandial vomiting of ingested matter. Associated with this, she has a history of bloating and belching, anorexia, and weight loss. Upon presentation, her vitals were in the normal range. Her BMI was 14.8 kg/m^2^. Otherwise, her physical examination was unremarkable. CBC, LFT, RFT, serum amylase, serum lipase, and stool examination were all normal. Abdominal CT scan identified decreased AMD (5 mm) and AMA (10°) (Figure 4). She was admitted with the diagnosis of SMA and prepared for surgery. Intraoperatively, a short SMA obstructing third part of duodenum was identified, and duodenojejunostomy was done. She was discharged on the 6^th^ postoperative day after she tolerated enteral feeding. On subsequent follow-ups, her symptoms were completely controlled, and she had gained 3 kg in weight.

## 2. Discussion

SMA syndrome is a rare condition mostly prevalent in cachexic patients. The medical conditions associated with catabolic states or rapid weight loss precipitate the syndrome [5]. The weight loss is coupled with subsequent loss of fat pad cushion that protects the SMA from the spine. As a result, the third portion of the duodenum is trapped in between the root of the mesentery anteriorly and the spine and the aorta posteriorly. The loss of fatty tissue results in the reduction of the normal AMA (38° – 65°) to less than 22° with concurrent reduction of AMD to below 8 mm [12]. Any other processes (spinal lordosis and short mesentery) that encroach in this space consequently narrow the space, and ultimately cause SMA syndrome through a relatively similar mechanism. In rare instances, the left renal vein might get externally compressed as it passes through the mesentery, resulting in nutcracker syndrome, as seen in case 2 [13].

As evident in all four cases, patients present with acute or chronic symptoms of duodenal obstruction including epigastric abdominal pain, early satiety, postprandial discomfort, nausea, and bilious emesis often after a meal, with associated bloating, belching, and reflux [8]. These symptoms are nonspecific and, hence, SMA syndrome is usually underdiagnosed or misdiagnosed with other anatomical or motility-related causes of the duodenal obstruction [14]. Symptoms mostly resolve following an episode of emesis. However, the anticipation of these symptoms makes them avoid eating altogether, further perpetuating a vicious cycle of weight loss and duodenal obstruction [6]. Postural relief of symptoms has been previously reported, as seen in case 1. In some cases, SMA syndrome might result in fatal complications, such as gastric dilatation [15] and acute upper intestinal ileus [16].

Upon clinical suspicion, imaging studies can help confirm the diagnosis. Traditionally, upper GI series was the mainstay of diagnosis [8]. A classic sign is a proximally dilated duodenum, with an abrupt termination at the third part of the duodenum with or without gastric distention [17]. However, it can give false negative results, if examined in the asymptomatic phase [18]. As in case 1, abdominal X-rays can identify complications, such as dilated stomach and proximal duodenum filled with gas and/or fluid [12, 14]. Abdominal CT imaging is the standard diagnostic modality. It can show acute angulation of the SMA and a reduced AMD [17]. An angle of less than 22° has a sensitivity of 42.8% and a specificity of 100% for SMA syndrome, whereas an AMD of less than 8 mm has a sensitivity and specificity of 100% [19]. Hence, the diagnosis in all four patients.

Conservative management with nutritional support is advocated initially to restore aortomesenteric fatty tissue [8]. Reconstitution of the fat pad re-tethers the SMA in its normal, lateral position alleviating the mechanical obstruction on the duodenum. However, close monitoring and correction of electrolytes during these early stages of nutritional support are mandatory. Complete obstruction can be treated initially by bowel rest, parenteral nutrition, or/and jejunal feeding, until enteral feeding is tolerated, while incomplete obstruction can be treated through progressive escalation from liquid meals to solid food through an oral feeding tube [20]. However, clear limits that define both the duration of conservative management and the minimal weight gain acceptable to alleviate these symptoms have not yet been established. As seen in cases 1 and 3, successful resolution of symptoms upon solely conservative management has been reported [7].

Due to the increased risk of surgical complications with malnutrition, surgical intervention is reserved upon failure of nutritional rehabilitation to resolve the symptoms [10]. Surgical options include open or laparoscopic duodenojejunostomy or duodenal mobilization and division of the ligament of Treitz (strong procedure) [8]. Even though the most recent standard operative treatment is laparoscopic duodenojejunostomy, open duodenojejunostomy can yield similar results as seen in cases 2 and 4. Despite being regarded as a safe and effective therapeutic approach, limitation of symptomatic improvement of surgical intervention has been seen in a 7-year follow-up study on 16 SMA patients treated with duodenojejunostomy [21, 22].

## 3. Conclusion

SMA syndrome remains a rare and often overlooked cause of proximal small bowel obstruction. This case series highlights the importance of maintaining a high index of suspicion, especially in young and cachectic patients. While conservative management with nutritional rehabilitation can lead to complete resolution in some patients, surgical intervention remains a vital option when symptoms persist. Early recognition and individualized management can lead to favorable outcomes and prevent unnecessary morbidity.

## Figures and Tables

**Table 1:** Summary of SMA Syndrome Cases Seen at Adera Medical and Surgical Center, 2024.

Sex	Age	BMI	Dominant symptom	Duration of symptom	WBC	Hgb	ESR	Serum electrolyte	AMA	AMD (mm)	Proximal GI dilation	Management	Symptomatic resolution
F	23	16 kg/m^2^	Vomiting	3 years	16,200	14	15	Mild hypokalemia (*K* + 3.0)	18°	7	Yes	Medical	yes
F	14	14.1 kg/m^2^	Vomiting	8 years	20,000	16	45	Normal	13°	7	Yes	Surgical	Yes
F	29	13.9 kg/m^2^	Vomiting	8 months	4600	14.3	2	Normal	15°	5	Yes	Medical	Yes
F	11	14.8 kg/m^2^	Vomiting	20 days	4200	17.3	10	Normal	10°	5	No	Surgical	Yes

## Data Availability

The data that support the findings of this study are available from the corresponding author upon reasonable request.

## Nomenclature

AMA: Aortomesenteric angle
AMD: Aortomesenteric distance
BMI: Body mass index
CBC: Complete blood count
CT: Computed tomography
ESR: Erythrocyte sedimentation rate
GI: Gastrointestinal
Hgb: Hemoglobin
LFT: Liver function test
OPD: Outpatient department
RFT: Renal function test
SMA: Superior mesenteric artery
u/L: Units per liter
WBC: White blood cell count.
